# Cultural Competence among Maternal Healthcare Providers in Bahir Dar City Administration, Northwest Ethiopia: Cross sectional Study

**DOI:** 10.1186/s12884-015-0643-8

**Published:** 2015-09-24

**Authors:** Amanu Aragaw, Tegbar Yigzaw, Desalegn Tetemke, Wubalem G/Amlak

**Affiliations:** Department of Reproductive health, College of Medicine and Health sciences, Bahir Dar University, Bahir Dar city, Ethiopia; Jhpiego- Ethiopia, Addis Ababa, Ethiopia; College of Health Sciences, Aksum University, Aksum, Ethiopia; Department of Nursing and Midwifery, Bahir Dar Health Science College, Bahir Dar, Ethiopia

## Abstract

**Background:**

Cultural competency is now a core requirement for maternal health providers working in multicultural society. However, it has not yet received due attention in Ethiopia. This study aimed to determine the level of cultural competence and its associated factors among maternal health care providers in Bahir Dar City Administration, Northwest Ethiopia.

**Methods:**

Institution based cross-sectional study was carried out using both quantitative and qualitative methods. Maternal health care providers from all health facilities were our study participants. Structured Questionnaire with some modification of Campinha Bacote’s tool was used to collect quantitative data from health workers and semi structured guide line was used for qualitative data among women. While quantitative data analysis was done using SPSS, qualitative data was analyzed using open code software. *P*-value of less than 0.05 was taken to determine statistical significance. Cronbach’s alpha was used to test internal reliability and a factor loading of 0.3 or greater was the criterion used to retain items.

**Result:**

Two hundred seventy four health workers and seven women were involved in the study. The overall competency level was 57.3 % thought vary in different subscales or stages. Of the cultural competent health workers near to three fourth (73.0 %) were in awareness stage which is the earliest stage of competence in which individuals were aware only their own culture but not the world view of their clients. The voices of mothers in the qualitative assessment also showed discordance in cultural competence with their healthcare providers. Female health workers almost six times [AOR,5.5; 2.71, 11.30] more competent than male providers and those who got in-service training related to maternal care provided services more culturally competent than their counter parts with [AOR,3.5; 1.4, 8.64]. Reliability Cronbach’s α coefficient value of cultural competence subscales showed 0.672,0 .719, 0.658, 0.714, and 0.631 for cultural awareness, knowledge, skill, encounter and desire, respectively.

**Conclusions:**

The overall competence level of health workers was low and the mean competence level falls in awareness stage in the continuum of culturally incompetent, culturally aware, culturally competent, and culturally proficient indicated that the providers were aware of only their own culture but not the world view of their clients. The voices of mothers also showed that they were dissatisfied for the services they got and the interactions they had with health care providers. Hence, we recommend on job training of health workers and incorporation of cultural components in the curriculum of health workers as it would be the key to provide culturally acceptable services.

## Background

Maternal mortality remains a major public health challenge. Globally, it is the leading cause of death among females aged 15–49 years old; 99 % of maternal deaths occur in developing countries [1, 2].

Ethiopia’s maternal morbidity and mortality rates are among the highest in the world [3] (Rob Stephenson: MaNHEP Base line report Part I, unpublished). Maternal mortality ratio is 676 per hundred thousand live births and 1 in 28 women have a lifetime risk of dying from pregnancy complications [5]. Each year, an estimated 22,000 women and 100,000 newborns die from complications related to childbirth. Most of these deaths occur at home due to lack of basic health care (Rob Stephenson: MaNHEP Base line report Part I, unpublished).

Utilization of maternity care services has paramount importance; it affects the well-being of the mothers as well as their children [1, 2]. This being the case, however, utilization of maternity care services in Ethiopia is extremely low even by the standards of most African countries (Ethiopian Society of Population Studies: Maternal health seeking behavior in Ethiopia, Unpublished). Recent data showed that Ethiopian antenatal coverage was 33.9 % and delivery service by trained health professional was only 10 % [5].

Practices during pregnancy and child birth are highly influenced by cultural values and beliefs [4] Culture is an integrated pattern of learned beliefs and behaviors that can be shared among groups. It includes thoughts, styles of communicating, ways of interacting, views on roles and relationships, values, practices, and customs [7]. Although the reasons for underutilization are many and complex, socio-cultural barriers need to be overcome if women are to access technical services and information that can prevent maternal mortality and morbidity [8]. A recent literature review on cultural barriers to seeking maternal healthcare services in Ethiopia concluded that cultural and religious beliefs affect maternal health service utilization (Rob Stephenson: MaNHEP Base line report Part I, unpublished).

A community based study done in Ethiopia recognized that women and their families were constrained by a number of factors which include the fact that important traditions and customs around birth were not recognized by health care providers[8]. Preference of birth attendants by known & trusted relatives or neighbors that are familiar with cultural context and norms; massage of the abdomen; squatting, kneeling and lying down position during delivery; ritual foods or drinks either during or after delivery; prayer or sprinkling of holy water as religious or spiritual elements are all among culturally recognized and valued components of safe childbirth by most Ethiopian women (Craig Hadley: MaNHEP Formative Research Report, unpublished).

Maternal health for Ethiopian women is surrounded with a number of cultural factors ranging from individual practices to institutional factors. In the first place, the traditional perception of pregnancy and childbirth is that of a natural condition, not requiring special health care. Women tend to be the major clients of the traditional healers and they then do not get proper medical care. Health facilities do not allow close relatives or friends to support the mother during labor (Craig Hadley: MaNHEP Formative Research Report, unpublished; and Charlotte W: Safe Motherhood Community-Based Survey, unpublished).

Child birth process in Ethiopia is viewed as a spiritual experience and the spiritual support provided by the mother of the woman is the most important support needed. Most of the women deferred the practice of religious rituals associated with childbirth during their hospitalization because they believed that the hospital environment was not conducive to these practices [10, 11].

Ethiopia is also home to diverse cultural and ethnic groups with implications for childbearing practices [12].

Cultural competence in health care describes the ability of systems to provide care to clients with diverse values, beliefs and behaviors, including tailoring delivery to meet their social, cultural, and linguistic needs [13, 14]. Cultural competence of healthcare providers is an educative process involving developing self-awareness, appreciating difference, valuing cultural practices other than one’s own, and acting flexibly in ways that accommodate these values [14].

Understanding clients’ beliefs can help providers align their services with their ideas or, when necessary, address local misconceptions. Providers can also bridge gaps by expressing respect for the clients’ beliefs and drawing connections between these beliefs and medical models of health [15]. On the other hand, lack of understanding and sensitivity to cultural beliefs and traditions on the part of providers can become barrier to use of maternal health services [16].

Despite its importance, to the best of our knowledge, there are no published studies on the subject in Ethiopia. Hence, we assessed cultural competence of maternal health care providers working in Bahir Dar City, Northwest Ethiopia.

## Methods

### Study design and area

Institution based cross-sectional study using quantitative and qualitative methods was conducted among health care providers in April 2012.

The study was conducted in Bahir Dar City, which is the capital city of the Amhara National Regional State, the second most populous region in the Federal Democratic Republic of Ethiopia. The city is sub divided in to 17 administrative kebeles (the smallest administrative units in Ethiopia) . The projected population number in 2012 was estimated to be 252,256, of whom 131,930 were females. The number of females in reproductive age group were 58,019, sharing 23 % of the total population [17].

### Study population and sampling

The quantitative study included health care professionals working in maternal care units (antenatal, labor and delivery, and postnatal clinics) at least for 6 months in all public and private healthcare facilities. Before the actual data collection census was conducted in each health care facility to identify potential healthcare professionals that could be study subjects. Based on our assessment 326 maternal health providers met the inclusion criteria and it was planned to include all in our study, but on the actual date of data collection 286 were available at their respective health facilities and participated in the study.

Qualitative data were collected from women who were attending antenatal services, mothers who had given birth and were in their waiting room, and women who came for postnatal visit. Women who had at least one antenatal contact with health care providers prior to the current visit and were believed to explain themselves and give rich information were purposively selected to participate in the study. Interviewing was continued until redundancy of ideas. Questions that reached saturation were removed and new questions were added whenever an information gap was identified and a total of seven women participated in the qualitative study.

### Data collection

Quantitative data were collected by interviewing using a structured cultural diversity questionnaire adapted from Campinha- Bacote’s model for cultural competency [14]. The instrument consisted of two sections: demographic characteristics of the healthcare providers and five point scale Likert type items intended to measure the respondents’ level of cultural competence [i.e., cultural awareness, cultural knowledge, cultural skills, cultural encounters and cultural desire]. The qualitative data were collected by conducting in-depth interviews of women who were exiting health facilities after receiving prenatal, delivery and postnatal services. We used a semi-structured interview guide to explore women’s perception of cultural, gender preference for maternal care services, advice they got from health care providers, cultural practices undertaken at home but prohibited in the health care institution, and to rate the overall management received.

## Operational definition

Cultural awareness is the lowest requirement in cultural competence. It is conducting self-examination of providers’ own biases towards women’s cultures and the in-depth exploration of one’s cultural and professional background [18].

Cultural skill refers to the ability of health care providers to conduct an assessment to collect relevant cultural data regarding the woman’s presenting problem as well as accurately conducting a culturally-based physical assessment [14].

Cultural encounter refers to directly engaging in face-to-face cultural interactions with clients from culturally diverse backgrounds in order to modify existing beliefs about a health related culture of women and to prevent possible stereotyping [14].

Cultural competence is an educative process that involves developing self-awareness, learning to appreciate difference, valuing cultural practices other than one’s own, and acting flexibly in ways that accommodate these values [14].

Scores for cultural competence of maternal healthcare providers were interpreted as follows: 91–100 % = culturally proficient; 75–90 % = culturally competent; 51–74 % = culturally aware; 25–50 % = culturally incompetent [7].

Cultural competent- health workers were labeled culturally competent if they answered ≥75 % of questions in favorable way.

Culturally incompetent: health workers were labeled culturally incompetent if they answered  <75 % of questions.

### Data analysis

The quantitative data were entered to Epi Info and exported to SPSS for analysis. Cultural competence on each sub scale was computed using IAPCC-R (Inventory for Assessing the Process of Cultural Competence among Health Care Professionals-Revised). The following response categories established by the researchers were used to interpret the responses: <1.50 = Strongly Disagree; >1.50 – 2.50 = Disagree; >2.50 – 3.50 = Undecided; >3.50 – 4.50 = Agree; >4.50 = Strongly Agree.

Both bivariate and multivariate analyses were done. All factors with a *p*-value <0.2 in the bivariate logistic regression analysis were further fit to multivariate logistic regressions for better prediction of determinants. The Hosmer-Lemeshow goodness-of-fit statistic was used to assess whether the necessary assumptions for the application of multiple logistic regression were fulfilled. Crude and adjusted Odds ratio with 95 % confidence intervals was computed. *P*-Value less than 0.05 were taken as significant. Cronbach’s alpha was used to test internal reliability of Likert types of items and a factor loading of 0.3 or greater was the criterion used to retain items.

Qualitative data analysis was done first by transcribing the tapes and translating the text from local language (Amharic) into English and subsequently conducting thematic analysis using the Open Code software.

## Ethical consideration

The study was ethically approved by research ethical committee review board of university of Gondar. Before Commencing data collection legal permission was obtained from officials of districts and health institutions. Moreover, all the study participants were informed about the purpose of the study and their right to refuse. Then consent (written for qualitative and oral for quantitative) was obtained from each study subjects. Strict confidentiality was assured through anonymous recording and coding and the questionnaire were kept locked.

## Results

### Quantitative result

#### Socio demographic characteristics

A total of 286 health workers [95.8 % response rate] were participated in the quantitative study. About half [50.7 %] were working in hospitals. The mean age was 30.34 [±6.64] years. Most study participants were nurses [74.8 %], females [60.2 %], Orthodox Christians [88 %] and of Amhara ethnic group [82.6 %] (Table 1).

**Table 1:** Socio demographic characteristics of maternal healthcare providers in Bahir Dar City Administration, April, 2012 (*n* = 274)

Socio-demographic characteristic	Number	Percent
1. Health facility		
• Hospital	139	50.7
• Health center	88	32.1
• Clinic	35	12.8
• Reproductive center	12	4.4
2. Gender		
▪ Male	109	39.8
▪ Female	165	60.2
3. Age		
▪ 20–29	154	56.2
▪ 30–39	91	33.2
▪ 40–49	25	9.1
▪ 50 ^+^	4	1.5
4. Place of birth		
▪ Urban	169	61.7
▪ Rural	105	38.3
5. Religion		
▪ Orthodox Christian	241	88
▪ Muslim	17	6.2
▪ Others	16	5.8
6. Ethnicity		
▪ Amhara	226	82.5
▪ Tigre	10	3.6
▪ Agaw	18	6.6
▪ Oromo	8	2.9
▪ Others	12	4.4
7. Mother tongue		
▪ Amharic	243	88.7
▪ Agewegna	14	5.1
▪ Others	17	6.2
8. Language other than first language and Amharic		
▪ Agewegna	7	2.6
▪ Oromifa	9	3.3
▪ Tigregna	15	5.5
▪ Others	10	3.6
9. Profession		
▪ Physician	15	5.4
▪ Midwives	42	15.3
▪ Health officer	12	4.4
▪ Nurse	205	74.8
10. Maternal care related In-service training		
-Yes	146	53.3
- No	128	46.7

### Cultural competence subscales

#### Cultural Awareness

Maternal health workers were asked 18 questions to explore their level of cultural awareness regarding the cultural requirement of the women they were serving and the result revealed that more than eight out of ten health workers (83.6 %) preferred non individualized, universal approach of management for all women regardless of residence, religion or other differences. More than six out of ten [62.9 %] of them agreed that as far as the service was given gender difference among healthcare providers had no impact on service utilization of women and, 34.3 %, agreed on excluding family members from delivery room for women in labor (Table 2).

**Table 2:** Cultural awareness of maternal health providers in Bahir Dar city Administration, April, 2012

	Agreement level					
	(*n* = 274)					
Awareness questions	Agree		Undecided		Disagree	
	Number	Percent	Number	Percent	Number	Percent
Pregnant women are prohibited from protein foods	180	65.7	8	2.9	86	31.4
Pregnant women are encouraged to work a lot to hasten the labor	202	73.7	9	3.3	63	23.0
Massaging the abdomen of laboring women is un common	57	20.8	13	4.7	204	74.5
I believe strictly on science rather than culture	193	70.4	16	5.8	65	24.1
Supernatural forces are responsible for pregnancy outcomes	223	81.4	12	4.4	39	11.2
Women’s perceptions of health are no different from my own	34	12.4	7	2.6	233	85.0
Husband &family members should be excluded from delivery room	94	34.3	7	2.6	173	63.1
The same approach should be followed when caring for all women	229	83.6	7	2.6	38	13.8
Am aware of the stereotyping attitudes towards my clients	180	65.7	17	6.2	77	28.1
Every laboring women must wear hospital gown	104	38.0	13	4.7	157	57.3
People from different cultural groups equally share the same values and beliefs to the host community	184	67.2	16	5.8	74	27.0
There are many differences in values and beliefs within any single ethnic group	267	97.4	2	0.7	5	3.0
Gender, age, class and generation are important in person’s identity	253	92.3	4	1.5	17	6.2
Cord cutting by the family themselves are important to individuals	33	12.0	10	3.6	231	84.4
Sex difference of healthcare provider has no impact on the service utilization of women	172	62.8	9	3.3	93	33.9
Roles for maternal health decisions vary between males and females in the community	241	88.0	5	1.8	28	10.2
To avoid imposing values on their clients practitioners should be aware of their own value and belief systems	261	95.3	3	1.1	10	3.6
Culture is influenced by personal, social and psychological factors	252	92.0	6	2.2	16	5.8

The items were checked for reliability using Cronbach’s alpha internal consistency coefficient and the overall alpha value was 0.672. The factor loading of the items retained ranged from 0.33 to 0.54. Three items, number 6, 7 and 10 as indicated in Table 2, which had rather item-total correlations less than 0.3 were removed from the sub scale.

The result of Inventory for Assessing the Process of Cultural Competence among Health Care Professionals-Revised [IAPCC-R] for this subscale revealed that 20 % were found to be culturally incompetent; 60.2 % were culturally aware; 17.2 % culturally competent and the 2.2 % were proficient.

#### Cultural knowledge

The result indicated that nearly 10 % of the health workers did not know well the greeting style of women they were serving, and similar proportion did not agree on the importance of knowing population percentages of the major ethnic groups, and on being familiar with key-words and phrases of women with limited proficiency in Amharic language (the working language) to adjust health care delivery. In addition, 28.8 % of the health workers did not know women’s perception and practice of discarding colostrums (first milk rich in antibodies and minerals that a mother’s breasts produce after giving birth) in their community. The Cronbach’s alpha value of items was 0.719 and only one item had corrected Item-total Correlation below the cutoff point.

The result of Inventory for Assessing the Process of Cultural Competence among Health Care Professionals-Revised [IAPCC-R] for this subscale showed that 10.2 % were found to be culturally incompetent; 49.6 % were culturally aware; 19.7 % culturally competent and the 20.4 % were proficient.

#### Cultural skill

More than one-fifth [21.2 %] of the participants did not allow a woman in labor for praying even if requested. Nineteen point seven percent of the health workers did not give chance for hygienic procedures to be taken by mothers or their families. One third (33.9 %) restricted mothers from back lying position during delivery, and 35 % allowed only delivery couches. More than three fourth [77 %] of health workers did not feel comfortable in asking client’s ethnic background.

Result of IAPCC-R of the subscale showed that 42.7, 28.1, 16.4, and 12.8 % of the participants were culturally competent, proficient, aware, and incompetent respectively. The reliability Cronbach’s alpha reading for elements was 0.658. All had Corrected Item total Correlation value above the cutoff point.

#### Cultural encounter

Near to 70 % (69.3 %) of the participants never asked to know the traditional needs of families or delivered women on burial of placenta before throwing away it.

Results of the IAPCC-R for the cultural encounter subscale found that 84.3 % were culturally incompetent, 12.0 % were aware, and only 1.8 % and 1.5 % were competent and proficient, respectively. This value of the items indicated that respondents were interacting with diverse community member occasionally or never at all. All the items had loading factor above 0.3 with Cronbach’s alpha 0 .714.

#### Cultural desire

Around 41 % of the respondents did not agree to incorporate culture to their clinical service. The result of IAPCC-R showed that 11.7, 33.9, 30.7, and 23.7 % of maternal health workers were culturally incompetent, aware, competent, and proficient respectively. The overall Cronbach’s Alpha level of items in this subscale was 0.631.

The result of cultural competence in the continuum of Campinha-Bacote’s Inventory for Assessing the Process of Cultural Competence-Revised (IAPCC-R) indicated that 9.1 % of maternal healthcare providers were culturally incompetent, 33.6 % aware, 47.1 % competent, and 10.2 % proficient. And, the overall cultural competence of the total study participants showed that 57.3 % of the respondents were culturally competent while the remaining 42.7 % were culturally incompetent. The mean score for the IAPCC-R was 73.0, which falls within the “culturally aware” category in the continuum of culturally incompetent, culturally aware, culturally competent, and culturally proficient.

Logistic regression analysis was conducted to identify predictors for cultural competence. Multivariate analysis found gender, place of birth; facility type and opportunity for in-service training were associated with cultural competence of maternal healthcare providers. Female health care workers were 3.3 times more likely to be competent than males and the association was also strongly significant [*p* = 0.003]. There was a positive association between competence level and place of birth [*p* = 0.009]. Health care providers who were born in rural area were 3.60 [95 % CI: 1.37, 9.40] times more likely to be culturally competent compared to their urban counterparts. Health care providers who worked in hospital were 63 % less likely to be culturally competent compared to those who worked in other health care facilities [AOR 0.37; 95 %, CI: 0.14, 0.97]. Health care providers who got in-service training related to maternal care were 3.5 times (1.40, 8.64) more likely to be competent than those who did not get the training [*p* = 0.007] (Table 3)

**Table 3:** The association of Socio demographic characteristics of maternal health care providers with cultural competence in Bahir Dar City Administration, 2012 (*N* = 274)

Socio demographic characteristics	Cultural competence		Crude OR	*P*-value
			(95 % CI)	AOR (95 % CI)
	# of competent	# of incompetents		
Type of health facility				
Hospitals	105	34	0.27(0.13,0.57)	0.04
Others	124	11	1.00	0.37 [.14, .97]
Place of birth				0.009
Urban	133	36	1.00	3.60(1.37, 9.40)
Rural	96	9	2.89 (1.33,6.27)	
Sex				0.003
Male	76	33	1.00	3.65(1.56, 8.50)
Female	153	12	5.54(2.71,11.33)	
Age				.24
20–35 years	184	37	1.00	1.96(.64, 6.10)
>35 years	45	8	1.13(0.49, 2.60)	
Ethnicity				0.000
Amhara	202	24	6.55(3.22,13.32)	16.13(4.28,60.84)
Others	27	21	1.00	
First language				0.09
Amharic	203	40	0.98 [.35,2.70]	0.24(0.05, 1.31)
Others	26	5	1.00	
Profession				0.09
Midwives	40	2	4.60(1.10, 19.56)	5,10(.74,35.17)
Others	189	43	1.00	
In-service training related to maternal care				0.007
Yes	92	36	5.96(2.74, 12.95)	3.48(1.40,8.64)
No	137	9	1.00	

### Qualitative result

Qualitative information about culturally competent care from their service providers was gathered from women who were receiving antenatal, delivery and postnatal care and the results are grouped into the following themes.

### Communication/ provider-client interaction

Majority of women in this study claimed that they did not receive warm greetings from healthcare professionals. Women also mentioned that health workers didn’t tell their health care roles in the institution and lack patience.

### Patient preferences and respect

Major complaint voiced by women who came for delivery service in this study was they were forced to deliver in the supine position. None of participants were asked about their favorite position from their health care providers. Even, those women who asked their preferred position to health workers blamed healthcare providers as unresponsive and rude with this regard. For example one participant expressed her sorrow for being prevented from her preferred position during delivery saying:

*“My preferred birthing position was kneeling, but since I am here in hospital, I was obliged to deliver in back lying position against my interest.” (25 years, merchant post natal mother)*

One of the aspects of maternity care about which women were most dissatisfied was that family members were not allowed to be present during birth

*As for the health workers, when you are in labor and get there (hospital), they start to shout at you as if you are a kid. But you know, your relatives are relatives, and as for traditional birth attendants, whether she knows you or not, she respects and communicates you politely. “(34 years, urban dweller during her ANC follow up)*

### Sex of health workers

Except for one study participant, all had no gender preference for the healthcare provider. They said that, as far as the service was given, they did not mind whether they were examined by males or females

### Diet advices and beliefs related to diet

Women were asked to express their beliefs whether there is a kind of food restriction during their pregnancy and the response indicated that larger proportion of them still believe in old unscientific tales. Foods like banana, mango, sugar cane and other sweet foods and fruits were not added to their regular diet as a way of safeguarding their lives and that of the unborn baby. Majority of women believed that eating such inhibited foods during pregnancy would cause prolonged labor, hypertension, and overweight new born. Despite these beliefs however, many of the study participants did not get appropriate advice about their diet during pregnancy.

## Discussion

There were no enough studies of the same objective to this particular study. In addition, consideration of varying sets of covariates did not allow for straight forward comparison of result between the different studies, even, in countries abroad. In view of this limitation, the findings of this particular research discussed as follows:

The result showed that the overall culturally competence level of maternal health workers was 57.3 %, even if the rate was not uniform across different constructs of the subscales. Using the IAPCC-R continuum of care 9.1 % of them were culturally incompetent, one third (33.6 %) were aware, 47.3 % competent, and the rest 10 % were proficient. This result was much better than a study done in USA in which 23 % of the health workers were culturally competent [19]. Possible explanations for this discrepancy might be related to the fact that many immigrant women from every continent with more diverse population could be in USA and so had more variation in socio-cultural contexts.

Furthermore, the mean score for the IAPCC-R was 73 which fall within the “culturally aware” category in the IACCP-R continuum. Also, as one goes from awareness to encounter stage of the subscale, competence level of participants decreased from 19.4 to 3.3 %. This might imply that while people were relatively aware of the importance of culturally competent care, its implementation with in or out of their profession and organization was very minimal. Nevertheless, it was promising that 54.4 % competence in the desire subscale means they did have the motivation to provide cultural competent care to their clients and would be one step to move to face the fact.

Literatures showed that clients taking part in the health care system are likely to look, think, and act, at least in some ways, differently from the health care professional [20]. However, in our study 12.4 % of health care providers mentioned that client’s perception about health was no more different from them. This might probably be due to the fact that formal cultural competent education was not given for health workers in the country.

The finding revealed that only 34.3 and 38.0 % of health providers were aware of the importance of support from respected family members in the delivery room and clothing preference of women (personal vs. hospital gown) respectively. In addition, 21.2, 19.7, and 35.4 % of the health workers respectively were disagreed for allowing praying; to take hygienic care by women themselves or their families as per their desire; to allow sleeping in normal than delivery couch for women in labor. This was substantiated by mothers who were involved in the qualitative study expressing unhappiness that the health care providers did not understand their cultural needs. This was in line with other studies in which clients reported that their healthcare providers did not appreciate their religious or cultural needs [16, 21, 22].

Experts considered providing client-centered health care on the basis of their interest as being able ‘to see through the patient’s eyes [9]. However, in this study one third of maternal health workers were not comfortable in assisting delivery in any position other than the medically accustomed supine position and this was supported by complaint voiced by women for the supine position they were forced to deliver. The result is not in agreement with the need of women in Ethiopia. Traditionally women in Ethiopia prefer kneeling position for the reason that this position hastens delivery and makes delivery less painful [16]. In this regard lack of courtesy of health workers in women’s position preference was identified as one of the barriers for institutional delivery that health workers should think over [16, 23, 24].

In our study 84 % of health providers preferred universal approach of management for all women seeking maternal services regardless of their residence, socio economic status, religious background or worldview. However, a study done in Ghana indicated that as there are a range of practices imbedded in each culture, maternal care providers should never assume that all women are the same as this results in stereotyping [21]. The gap of our study with this fact might be related to lack of culturally tailored curriculum of health workers or lack of awareness of providers on the impact of culture on maternal health services.

Gender difference between the provider and the client can act as a barrier in the use of health services [21, 25] About 63 % of health workers participated in this study agreed that being a male or female health care provider had no impact on the service utilization of women as far as the service was given. This was also supported by the result of qualitative part in which majority of study participants had no gender preference for the healthcare provider. This result is not in agreement with other studies conducted in Ethiopia and other parts of the world [22, 23, 26, 27] where women preferred to be seen by female care providers and stressed that having a female physician made them more comfortable, especially for gynecological matters. The gap might be attributed to small sample size in our study in relation to large scale studies at region or country level in other studies.

The major predictors identified for cultural competence in the logistic regression model were: the organization type in which health professionals work, birth place, gender, and in-service training.

Health care providers working in hospital were 63 % less likely to be culturally competent compared to those who worked in other health care facilities [AOR 0.37; 95 %, CI: 0.14, 0.97] and the association was also significant [*p* = 0.03]. This could be explained by the fact that hospital workers usually gave service for referred cases that came out of their catchments and probably women from societies of diverse ethnic groups, language, and cultures in comparison to health centers and clinics that serve communities with their nearby surroundings that they are familiar.

Healthcare providers who were born in rural area were 3.7 [95 % CI: 1.37, 9.40] times more likely to be culturally competent compared to their urban counterparts. This might be related to the fact that rural community retain and continue their ancestor distinct traditional practices than urban dwellers in which the latter have been more likely to adopt the western cultural habits and customs as they are more liable to be influenced by media, technology and other aspects of globalization.

Female health workers were 3.65 times, [95 % CI: 1.56, 8.50] more likely to be culturally competent compared to men. The association was also strongly significant [*p* = 0.005]. Like other parts of Ethiopia in the study area too, most of traditional practices were expected to be practiced by and/or practiced on women for whom females might probably got to share or at least observed cultural practices in their families and neighbors.

Health care providers who got in-service training related to maternal care were 3.5 times (95 % CI 1.40, 8.64) more likely to be competent than those who did not get the training [*p* = 0.007]. This might indicate that competence could be brought by training of health workers as they might get chance of acquisition of up-to-date information from specifically designed maternal care services.

However, in the present study there was no significant relationship between general cultural competences and the level of academic credentials.

## Strength and weakness

The study tried to investigate common but unforeseen barrier of health seeking for maternal healthcare.

As limitation there could be a possibility of social desirability bias. Also, since there were no enough studies of the same objective to this particular study, there were no appropriate comparisons. In addition Consideration of varying sets of covariates did not allow for straight forward comparison of result between the different studies, even, with the results of countries abroad.

## Conclusion

The overall competence level of maternal health workers was low and on average the level falls in Cultural awareness stage, second stage, indicated that health workers consider this issue important but it requires them to become culturally competent and culturally proficient.

The voices of mothers in the qualitative assessment also showed that they were dissatisfied for the services they got and the interactions they had with health care providers. Mothers, especially after delivery, also mentioned that they were inhibited from activities that could be freely practiced if they were at their homes.

Organization type, birth place, Gender, and in-service training were among major predictors identified for cultural competence. Working other than hospital, grew in rural community, being Amhara and those maternal health workers who got in service training respectively were more competent than their counterparts.

These results have national implications for considering client’s varying degrees of religious, ethnic and other cultural adherences in maternal services. The findings of this study will also encourage continuing research, education, and culturally sensitive interventions as part of the health worker’s mission to provide holistic care.

Hence, we recommend in designing culture based trainings or curriculum based education for maternal health workers and to undertake community assessment to identify common cultural practices and shape services accordingly. Finally, heterogeneous ethnic backgrounds and cultures found in the Ethiopian population, future large scale studies on health providers’ cultural competence toward various cultural groups is forwarded.
